# Regulation of cyp26a1 on Th17 cells in mouse peri-implantation

**DOI:** 10.1111/jcmm.12196

**Published:** 2013-12-10

**Authors:** Hai-Yan Liu, Huhe Chao, Zhen-Kun Liu, Hong-Fei Xia, Zhihui Song, Ying Yang, Jing-Pian Peng

**Affiliations:** a State Key Laboratory of Reproductive Biology, Institute of Zoology, Chinese Academy of SciencesBeijing, China; b University of Chinese Academy of SciencesBeijing, China

**Keywords:** cyp26a1, Th17, pregnancy, RORγt, RA, RARα

## Abstract

Cytochrome P450 26A1 (cyp26a1) is expressed in the mouse uterus during peri-implantation. The repression of this protein is closely associated with a reduction in implantation sites, suggesting a specific role for cyp26a1 in pregnancy and prompting questions concerning how a metabolic enzyme can generate this distinct outcome. To explore the effective downstream targets of cyp26a1 and confirm if its role in peri-implantation depends on its metabolic substrate RA (retinoic acid), we characterized the changes in the peripheral blood, spleen and uterine implantation sites using the cyp26a1 gene vaccine constructed before. Flow cytometry results showed a significant increase in CD4^+^RORγt^+^ Th17 cells in both the peripheral blood and spleen in the experimental group. The expression of RORγt and IL-17 presented the Th17 cells reduction in uterus followed by the suppression of cyp26a1 expression. For greater certainty, cyp26a1 antibody blocking model and RNA interference model were constructed to determine the precise target immune cell group. High performance liquid chromatography results showed a significant increase in uterine at-RA followed by the immunization of cyp26a1 gene vaccine. Both the ascertain by measuring RARα protein levels in peri-implantation uterus after gene vaccine immunization and researches using the specific agonist and antagonist against RARα suggested that RARα may be the main RA receptor for signal transduction. These results provided more evidence for the signal messenger role of RA in cyp26a1 regulation from the other side. Here, we showed that the cyp26a1-regulated Th17 cells are dependent on at-RA signalling, which is delivered through RARα in mouse peri-implantation.

## Introduction

As a metabolic enzyme of the vitamin A metabolite retinoic acid (RA), cytochrome P450 26a1 (cyp26a1) is a member of the cyp26a family [1–2]. The cytochrome P450 (CYP) family is a superfamily of haem-binding monooxygenases that catalyses numerous important biological reactions, like non-specific oxidative conversions of many steroids, lipids, and a variety of xenobiotics and environmental toxins [2]. This superfamily is so large that contains at least 74 families and each family is constituted by a plurality of family members. These family members are distinct from their particular distribution, biologically active pattern or preferred substrates. Cyp26a1 is expressed in the female reproductive tract [4, 5] and takes all-trans-RA (at-RA) as its preferred substrate. Our previous work shown that the significant up-regulation of cyp26a1 in the endometrial epithelial cells of mice on D5 and D6 of gestation [6], the key time for embryo implantation, suggested its important role in peri-implantation. Other studies also showed that the expression of cyp26a1 in endometrial epithelial cells is regulated through progesterone, but is not significantly influenced through the co-administration of oestrogen [5]. Interference to cyp26a1 could resulting in serious consequences of pregnancy; the implantation sites number was significantly reduced after the intrauterine injection of cyp26a1-specific antisense oligos or anti-cyp26a1 antibody on D3 of pregnancy [6]. Accordingly, the expression of cellular retinoic acid–binding protein 1 and tissue trans glutaminase was markedly increased in the uterine luminal epithelium after the intrauterine injection [6]. These researches prompted that the cyp26a1 role in successful pregnancy can never be overlooked, whereas the specific mechanism involving cyp26a1 and foetal loss remains unclear.

Retinoic acid (RA) is the substrate of cyp26a and also a largely used medicine in clinic. It has multiple isomers like 13-cis-RA, 9-cis-RA and at-RA. Among them, at-RA is the preferred substrate of cyp26a1 and the potential inducer of cyp26a1 expression in the liver [1, 2, 7, 8]. As the active metabolite of vitamin A, RA is a crucial factor for embryonic development [9], meiotic initiation [10] and the regulation of immunity [11]. Recent studies have shown that RA is also essential for the development of certain immune cell subtype. Mucida *et al*. identified the vitamin A metabolite retinoic acid as a key regulator of TGF-β–dependent immune responses, which inhibits the IL-6–driven induction of pro-inflammatory Th17 cells [12]. It is already known that mammalian immune system is remodelled during peri-implantation to tolerate paternal alloantigens and simultaneously protect the female parent from infections for successful pregnancy. T-helper (Th) cells play a central role in this process [13, 14].

On the basis of this information,we speculated that Th17 cells may involved in the role of cyp26a1 during peri-implantation. However, the certainty of this and mechanism underlying needs further research. The aim of this study was to identify the precise target immune cell subset of cyp26a1 in peri-implantation and explore if the role of cyp26a1 depends on RA.

Here, we used a cyp26a1 gene immunization model, which has been proven to make enormous impacts on mouse pregnancy to explore the target cell group of cyp26a1 in peri-implantation. Moreover than this, for greater certainty, cyp26a1 antibody blocking model and RNA interference model were constructed to determine the precise target immune cell group. High performance liquid chromatography (HPLC) was employed to assess the uterine content of RA for further determination if the RA is necessary in cyp26a1 regulation. Both the ascertain by measuring RARα protein levels in uterus and researches using the specific agonist and antagonist against RARα suggested that RARα may be the main RA receptor for signal transduction. Moreover than this, it provided more evidence for the signal messenger role of RA in cyp26a1 regulation. These observations suggested that RA is an essential mediator in cyp26a1 regulation and Th17 cells are an important Th cell subtype of cyp26a1 targeting in mice peri-implantation.

## Materials and methods

### Mouse mating strategy

Sexually mature and healthy Kunming white (KM) mice were purchased from the Experimental Army Laboratory Animal Resources Center. The mice were housed in a temperature-and humidity-controlled room with a 12-h light/dark cycle. This study was conducted in strict accordance with the recommendations of the Institutional Animal Care and Use Committee of the Institute of Zoology, Chinese Academy of Sciences. All protocols were approved through the Use Committee of the Institute of Zoology, Chinese Academy of Sciences. All surgery was performed under sodium pentobarbital anaesthesia, and all efforts were made to minimize suffering. Female mice were caged overnight with male mice of the same strain, and the presence of a vaginal plug on the next morning was considered as day 1 of pregnancy (D1).

### Plasmid construction and animal immunization

Full-length rat cyp26a1 cDNA was cloned from the uteri of pregnant rats, using specific primers with HindIII/XhoI restriction sites (forward primer: 5'CGAAGCTT (HindIII) ATGGGGCTCCCGGCGCTGCT3'; reverse primer: 5'CGCTCGAG (XhoI) TCAGATATCTCCCTGGAAGTGG3'). The PCR products were purified and cloned into the pGEM-T vector (Promega, Madison, WI). Both pGEM-T-cyp26a1 and the pCR3.1 vector (Invitrogen, Eugene, OR) were digested with HindIII/XhoI (Promega) at 37°C for 2 h, and subsequently, the fragment was ligated into pCR3.1 using T4 ligase (Promega) at 16°C overnight to construct pCR3.1-cyp26a1. HindIII/XhoI were used to digest the recombinant plasmid pCR3.1-cyp26a1 at 37°C for 2 h, and the insert fragment was confirmed through sequencing [6]. The expression of this recombinant plasmid was examined *in vitro*, as previously described.

Divided the mice into three groups: one group was immunized in the thigh muscle using 100 μl of saline containing 20 μg of pCR3.1-cyp26a1 per mouse, and the other two groups were immunized with either 100 μl of saline or 100 μl of saline containing 20 μg of empty pCR3.1 plasmid in the thigh muscles of mice as a control group. The immune effect was strengthened threefold through injection every 7 days. At 3 days after the last immunization, the female mice were coupled with male mice at a ratio of 2:1 until vaginal plugs were observed. All female mice were completely coupled within 2 weeks. The mouse uterus were excised from D4 to D7 pregnancy mice and frozen in liquid nitrogen for further analyses.

### Reagents administration

Anti-cyp26a1 antibody was administrated by intrauterine injection. The surgeries were performed on D3 of pregnancy. 10 μl (1 mg/ml) antibody was injected into each side uterine horn, respectively, as antibody treatment group. Same volume intrauterine injections of PBS and rabbit IgG were for control groups. These mice were killed on D5 of pregnancy. Uterus were excised and frozen in liquid nitrogen for further protein analysis.

Lentiviral interference segments of cyp26a1 (NO. 306, Genepharma, China) were administered through tail vein injection at 100 μl per mouse on D3 of pregnancy. Use same volume unrelated fragments (NC) administration as control group. These mice were killed on D5 of pregnancy. Uterus were excised and frozen in liquid nitrogen for further protein analysis.

The RARα agonist (Am-580, CAS NO: 102121-60-8, Enzo) and its antagonist (Ro-41-5253, CAS NO: 71441-28-6, Enzo) were separately administered through tail vein injection (n = 3 for each group) at 125 μg per mouse in volume of 100 μl on D3 of pregnancy. Use same volume mixed solvent tail vein injection, DMSO: saline as 1: 3, as control. These mice were killed on D5 of pregnancy. Uterus were excised and frozen in liquid nitrogen for further protein analysis.

### Western blotting

Mouse uterine protein was extracted from implantation sites by non-denaturing lysis buffer and protein concentrations were determined by Bio-Rad protein assay (Bio-Rad). Uterine proteins were separated on 12% SDS–PAGE and electroblotted onto a nitrocellulose membrane (Pall, New York, NY). After blocking in 5% non-fat milk at 37°C for 1 h, the membranes were incubated with primary antibody at 4°C overnight. Membrane was washed in TBST buffer gently. Incubate the membranes with corresponding secondary antibody at 37°C for 1 h. Then, wash the membranes in TBST thoroughly. Chemiluminescence reaction (Pierce, Rockford, IL) was performed to test each protein. The bands were analysed using the Quantity One analysing system (Bio-Rad).

Here are the primary and secondary antibodies list used in western blotting: anti-cyp26a1 (CYP26A12-A, Acris, Hiddenhausen, Germany), anti-RORγt (17-6988, eBioscience, USA), anti-IL-17 (sc-7929, Santa Cruz, USA),anti-RARα (sc-15040, Santa Cruz, USA); goat anti-rat (112-035-003, Jackson ImmunoResaerch, USA), goat anti-rabbit (074-1506, KPL, USA), rabbit anti-goat (14-13-06, KPL, USA).

### Immunohistochemistry

The frozen sections (8 μm) of mouse uterus implantation sits were mounted on 3-aminopropyltriethoxy-silane–coated slides and fixed in 4% PFA for 10 min. After washing the sections with PBS, endogenous peroxidase activity was blocked by 0.3% H_2_O_2_ for 10 min at room temperature. After blocking in horse serum for 1 h at 37°C, the sections were incubated with first antibody that diluted in PBS overnight at 4°C. Normal rat IgG was used as a negative control. After washing thoroughly in PBS, the sections were incubated with a secondary antibody (112-035-003, Jackson ImmunoResearch, USA) diluted 1:400 in PBS at 37°C for 2 h. The colour was developed using diaminobenzidine tetrahydrochloride (Sigma–Aldrich, USA). The sections were counterstained with haematoxylin (Sigma–Aldrich, USA). Here is the primary and secondary antibodies list used in immunohistochemistry: anti-RORγt (17-6988, eBioscience, USA), anti-IL-17 (sc-7929, Santa Cruz,USA), anti-cyp26a1 (CYP26A12-A, Acris, Hiddenhausen, Germany), anti-RARα (sc-15040, Santa Cruz, USA); goat anti-rat (112-035-003, Jackson ImmunoResaerch, USA), goat anti-rabbit (074-1506, KPL, USA), rabbit anti-goat (14-13-06, KPL, USA).

### Isolation of Mononuclear Cells from spleen and peripheral blood

Blood was drawn from the eyeballs and diluted with a volume of onefold Hank's solution for mononuclear cell isolation. Isolation was performed with Histopaque®-1083 (Sigma-Aldrich, USA) and centrifugation at 200 g for 30 min. The spleen of the same mice was also used for splenocyte isolation. Use core needle grinding to obtain splenic cells, then splenocytes were also isolated using Histopaque®-1083 (Sigma-Aldrich, USA) and centrifugation at 200 g for 30 min. Fresh peripheral blood was collected from D4 to D7 of pregnancy for flow cytometry analysis no more than 4 h later. Spleens were excised from D4 to D7 of pregnancy for further flow cytometry analysis, also no more than 4 h later.

### Flow cytometry analysis

Surface staining was performed for 15-20 min with fluorescently labelled CD4 antibody (11-0041, eBioscience, USA). After surface staining, the cells were resuspended in fixation/permeabilization solution (88-8823-88, eBioscience, USA), and intracellular staining of RORγt (17-6988, eBioscience, USA) was performed according to the manufacturer's protocol. Flow cytometry analysis was performed on FACS Calibur (BD Biosciences) instruments and analysed using FlowJo software 8.7 (Tree Star Inc.).

### High performance liquid chromatography

The experimental approach of RA level assay in uterus tissue was based on the classic method of RA measuring [15, 16] and improved according to the experimental conditions. The whole uterus was lysed in physiological saline (1:20) to obtain tissue lysate and then the tissue solution was centrifuged to remove the precipitate. The impurities were extracted from this tissue solution using N-hexane (10:1) and 0.025 mol/L NaOH-carbinol (10:6). N-hexane (10:1) and 4 mol/L HCl (1:5) were added to extract RA from the samples. The exsiccation of the supernatant was performed at a low temperature (4°C), and the samples were dissolved in acetonitrile for HPLC analysis. All laboratory manipulations involving RA were performed in a darkened room under dim yellow light to avoid RA isomerization. The HPLC detection was performed at the Protein Research Platform, Institute of Biophysics at the Chinese Academy of Sciences. The protein separation was performed with a reverse phase C18 column (250 mm×4.6 mm I.D., 5 m, Welch Materials, Inc.) eluted using a mixture of acetonitrile *versus* 1% acetic acid (86:14) at a flow rate of 1 ml/min under a 350 nm detection wavelength at an ambient temperature of 25°C. The different RA isomers were separated at different elution times. The elution gradient was 14 min (13-cis-RA)-16.25 min (9-cis-RA)-17 min (at-RA). The HPLC analysis was performed with a Dionex Ultimate U3000 HPLC device (Dionex, Sunnyvale, USA). The RA concentration was analysed using Chroméléon software (Dionex, Sunnyvale, USA).

### Statistical analysis

All results were reported as the mean ± SEM or the mean ± SD. One-way ANOVA or a paired t-test was used to assess the significance of differences. A value of p < 0.05 represented statistical significance, and p < 0.01 represented sufficient statistical significance. The software used for statistical analysis was SPSS 15.0 (SPSS Software, Chicago).

## Results

### By the immunization of pCR3.1-cyp26a1 gene vaccine, a significant reduction on cyp26a1 was appeared at uterine implantation sites during mice peri-implantation

**Figure 1:**
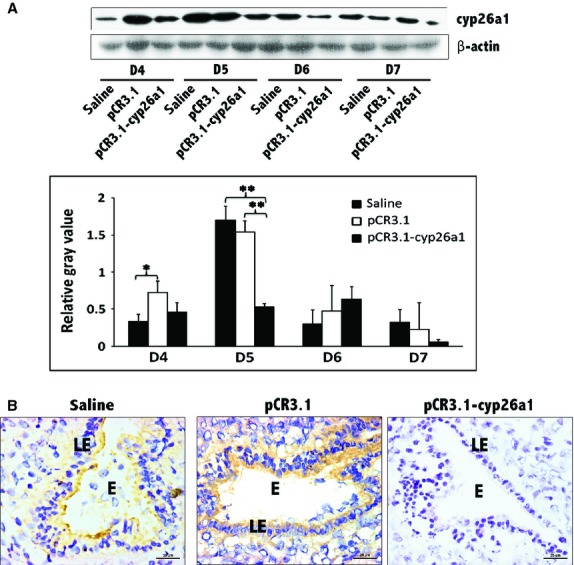
Decrease in cyp26a1 levels at uterine implantation sites following pCR3.1-cyp26a1 immunization during mice peri-implantation. (A) cyp26a1 levels were significantly reduced at uterine implantation sites based on Western blotting, especially on D5 of mice pregnancy. β-actin was used as a loading control. The bars represent the SD of the mean of the relative value in grey (cyp26a1/β-actin). A paired *t*-test was used to assess the significance of differences. Pairwise comparisons between each treatment group, *P < 0.05, **P < 0.01. At least, three independent experiments were repeated at each time-point. A total of 36 samples from pregnant mice were assessed. (B) Cyp26a1 levels were obviously reduced at implantation sites of D5 pregnancy based on immunohistochemistry; bars = 25 μm. Three independent experiments were repeated for this time-point. A total of nine samples from pregnant mice were assessed. E, embryo; LE, Luminal epithelium.

To examine the role of cyp26a1 in mice implantation, we previously generated a recombinant plasmid pCR3.1-cyp26a1 using cyp26a1 cDNA cloned from the rat uterus. The use of DNA vaccines to examine reproduction has been confirmed in previous reports [17, 18]. Heterogeneous antigens did not induce host immune tolerance and did not elicit cytotoxic responses that might result in abnormal reproductive function or other damage [19]. By the inhibition of pCR3.1-cyp26a1 gene vaccine, the pregnancy rate of the mice immunized with recombinant plasmid pCR3.1-cyp26a1 was significantly reduced [6]. As indicated in Figure 1, cyp26a1 was decreased significantly at uterine implantation sites, either based on Western blotting or immunohistochemistry. The most significant reduction in cyp26a1 appeared in D5 pregnancy (Fig. 1), the key time for embryo implantation and also the peak point of cyp26a1 expression in healthy pregnancy.

### Th17 cell increased in the peripheral blood and the spleen, while reduced in uterine implantation sites following pCR3.1-cyp26a1 immunization during peri-implantation

To explore if certain subtype of immune cell would involve in the foetal loss induced by pCR3.1-cyp26a1 immunization, Th17 cells have been tracked in peripheral and uterus.

**Figure 2:**
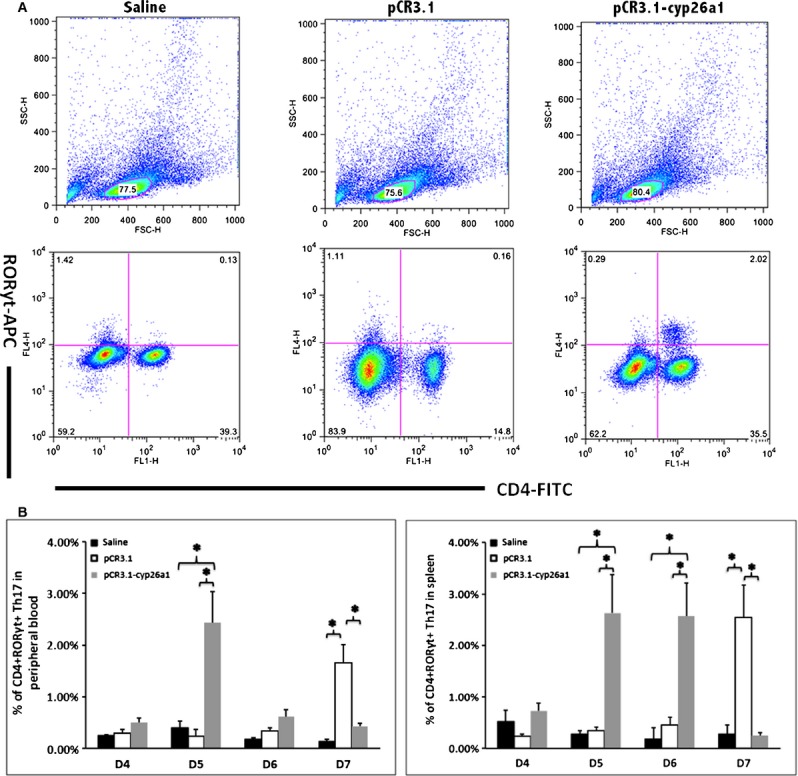
Th17 cell increased in the peripheral blood and the spleen following pCR3.1-cyp26a1 immunization during mice peri-implantation, especially on D5 of mice pregnancy. (A) Flow cytometry scatterplot of Th17 cells. Th17 cells are CD4^+^ RORγt^+^. (B) The Th17 cell proportion increased significantly in the peripheral blood and the spleen under the influence of recombinant plasmid, especially on D5 of pregnancy. The CD4^+^ RORγt^+^ Th17 cell ratio was evaluated by flow cytometry analysis. The data are expressed as the% of CD4^+^ RORγt^+^ double positive cells. The bars represent the SD of the mean. A paired *t*-test was used to assess the significance of differences. Pairwise comparisons between each treatment group, *P < 0.05. At least, three independent experiments were repeated for each time-point. A total of 49 samples from pregnant mice were assessed.

**Figure 3:**
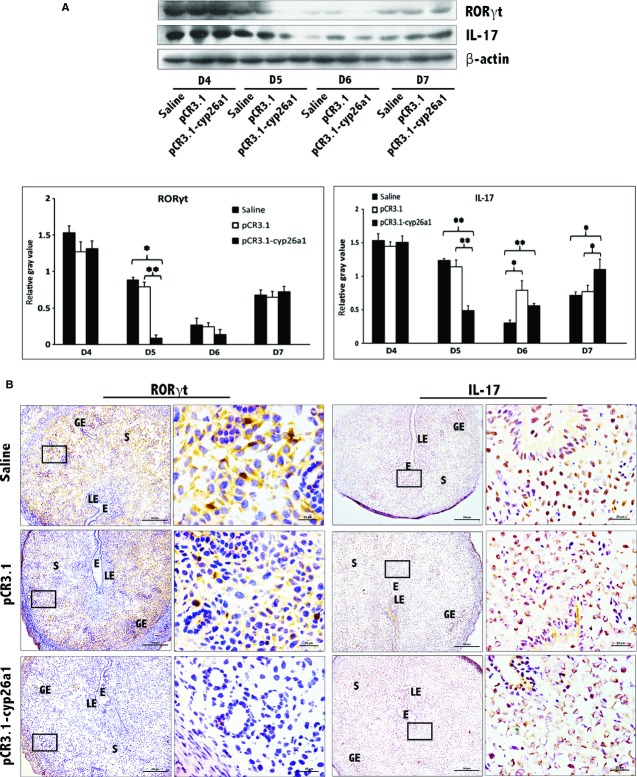
Decrease in Th17 cells at uterine implantation sites following pCR3.1-cyp26a1 immunization during mice peri-implantation. (A) Th17 levels were significantly reduced at uterine implantation sites based on Western blotting, especially on D5 of mice pregnancy. RORγt is the specific marker of Th17 cell, IL-17 is the specific cytokines secreted by Th17 cells. β-actin was used as a loading control. The bars represent the SD of the mean of the relative value in grey (RORγt/β-actin or IL-17/β-actin). A paired *t*-test was used to assess the significance of differences. Pairwise comparisons between each treatment group, *P < 0.05, **P < 0.01. At least, three independent experiments were repeated for each time-point. A total of 36 samples from pregnant mice were assessed. (B) Th17 levels were obviously reduced at implantation sites of D5 pregnancy based on RORγt and IL-17 immunohistochemistry stain; bars = 200 and 25 μm. At least, three independent experiments were repeated for this time-point. A total of nine samples from pregnant mice were assessed. E, embryo; S, uterine stroma; LE, Luminal epithelium; GE, Glandular epithelium.

Flow cytometry was employed to analyse the Th17 cell ratio in peripheral blood and spleen (Fig. 2). In those gene vaccine–immunized mice, Th17 subpopulation expansion could be clearly observed in peripheral blood and spleen during peri-implantation. To gain insight into the effect of the recombinant plasmid on Th17 cells in the decidua, we performed immunohistochemical localization and western blotting analysis on RORγt, the specific transcription factor of Th17 cell and IL-17, the main function cytokine that Th17 secrete (Fig. 3). Immunohistochemical localization shows the decrease in RORγt and IL-17 in implantation sites (Fig. 3B). Also, these observations were confirmed by the results of western blotting (Fig. 3A). Highly similar trends of both RORγt and IL-17 further confirmed that Th17 cells were significantly reduced in uterine implantation sites after the immunization of anti-cyp26a1 gene vaccine. The most significant fluctuations in Th17 cells occurred on D5, the same time as the most serious reduction in cyp26a1.

### Th17 cells changes in cyp26a1 antibody blocking model and cyp26a1-lentiviral interference model

**Figure 4:**
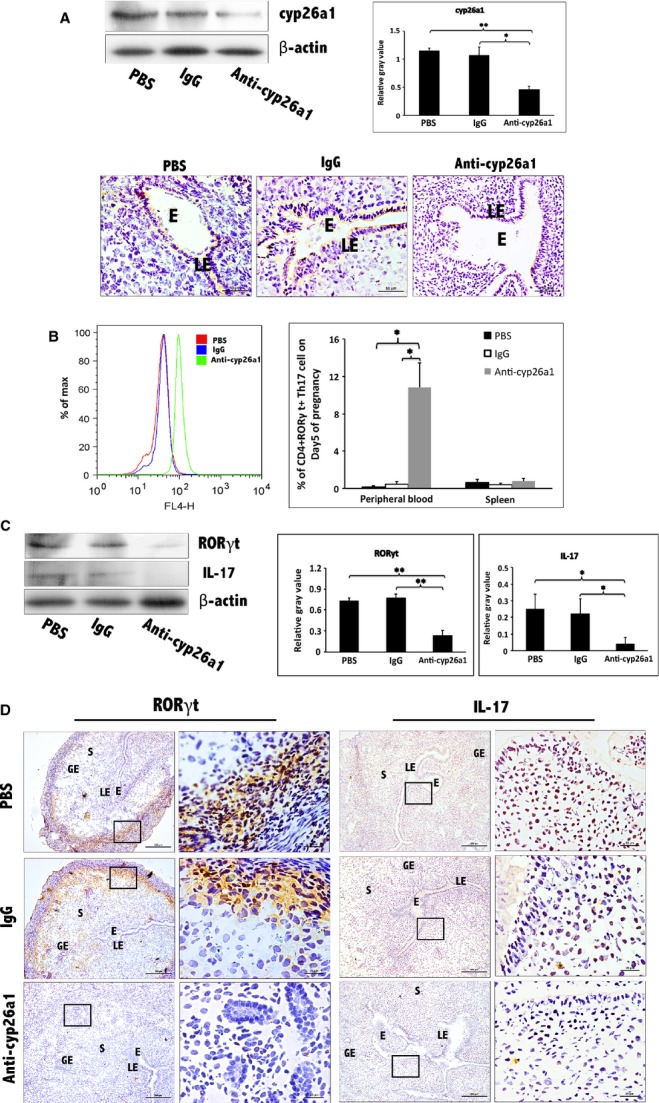
Further certainty, the regulation of cyp26a1 on Th17 cells at uterine implantation sites by uterine horn injection of anti-cyp26a1 antibody during mice peri-implantation. (A) Decrease in cyp26a1 levels at D5 uterine implantation sites following uterine horn injection of cyp26a1 antibody on D3 of pregnancy. Cyp26a1 levels were reduced at uterine implantation sites based on Western blotting and immunohistochemistry. β-actin was used as a loading control. The bars represent the SD of the mean of the relative value in grey (cyp26a1/β-actin). A paired *t*-test was used to assess the significance of differences. Pairwise comparisons between each treatment group, *P < 0.05, **P < 0.01; bars = 50 μm. The data were derived from three separate samples from pregnant mice. At least, three independent experiments were repeated for this time-point. A total of nine samples from pregnant mice were assessed. (B) Th17cell increased in the peripheral blood and the spleen following uterine horn injection of cyp26a1 antibody. Flow cytometry histogram of Th17 cells is on the left side. Th17 cells are CD4^+^ RORγt^+^. Th17 cell ratio was evaluated by flow cytometry analysis. The data are expressed as the% of CD4^+^ RORγt^+^ double positive cells. The bars represent the SD of the mean. A paired *t*-test was used to assess the significance of differences. Pairwise comparisons between each treatment group, *P < 0.05. Three independent experiments were repeated for this time-point. A total of nine samples from pregnant mice were assessed. (C) Th17 levels were significantly reduced at uterine implantation sites based on Western blotting of pregnancy D5. β-actin was used as a loading control. The bars represent the SD of the mean of the relative value in grey (RORγt/β-actin or IL-17/β-actin). A paired *t*-test was used to assess the significance of differences. Pairwise comparisons between each treatment group, *P < 0.05, **P < 0.01. The data were derived from three separate samples from pregnant mice. Three independent experiments were repeated for this time-point. A total of nine samples from pregnant mice were assessed. (D) Th17 cell levels were obviously reduced at implantation sites of D5 pregnancy based on immunohistochemistry of RORγt and IL-17; bars = 200 and 25 μm. The data were derived from three separate samples from pregnant mice. At least, three independent experiments were repeated for this time-point. A total of nine samples from pregnant mice were assessed. E, embryo; S, uterine stroma; LE, Luminal epithelium; GE, Glandular epithelium.

**Figure 5:**
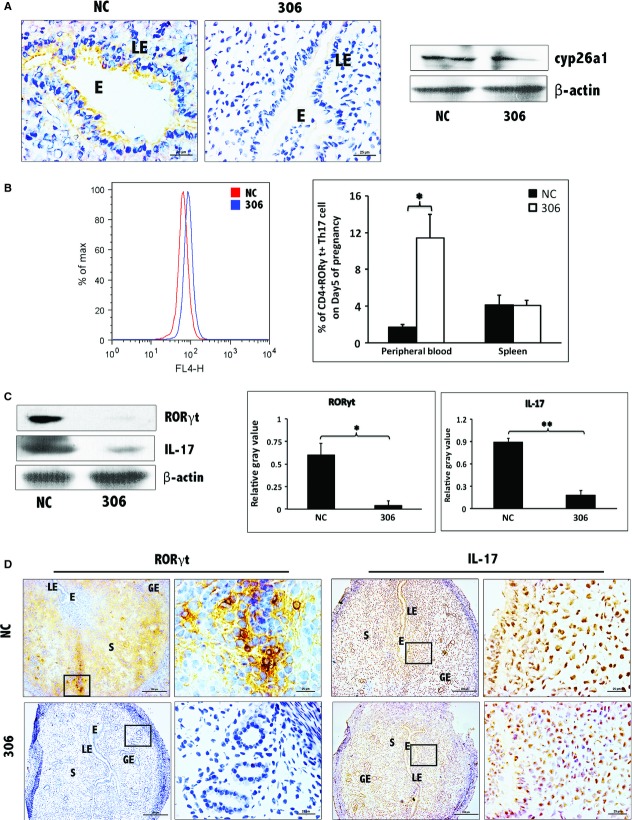
Further certainty, the regulation of cyp26a1 on Th17 cells at uterine implantation sites by tail vein injection of cyp26a1 lentiviral interference segment (No. 306) during mice peri-implantation. (A) Decrease in cyp26a1 levels at uterine implantation sites of D5 following tail vein injection of cyp26a1 lentiviral interference segment (306) on D3 of pregnancy. Cyp26a1 levels were reduced at uterine implantation sites based on Western blotting and immunohistochemistry; bars = 25 μm. The data were derived from two separate samples from pregnant mice. At least, three independent experiments were repeated for this time-point. A total of six samples from pregnant mice were assessed. (B) Th17 cell increased in the peripheral blood, but the spleen following tail vein injection of cyp26a1 lentiviral interference segment (No. 306). Flow cytometry histogram of Th17 cells is on the left side. Th17 cells are CD4^+^ RORγt^+^. Th17 cell ratio was evaluated by flow cytometry analysis. The data are expressed as the% of CD4^+^ RORγt^+^ double positive cells. The bars represent the SD of the mean. A paired *t*-test was used to assess the significance of differences. Pairwise comparisons between each treatment group, *P < 0.05. Three independent experiments were repeated. A total of nine samples from pregnant mice were assessed. (C) Th17 levels were significantly reduced at uterine implantation sites based on Western blotting of D5 pregnancy. β-actin was used as a loading control. The bars represent the SD of the mean of the relative value in grey (RORγt/β-actin or IL-17/β-actin). A paired *t*-test was used to assess the significance of differences; *P < 0.05, **P < 0.01. The data were derived from two separate samples from pregnant mice. Three independent experiments were repeated for this time-point. A total of six samples from pregnant mice were assessed. (D) Th17 levels were obviously reduced at implantation sites of D5 pregnancy based on immunohistochemistry of RORγt and IL-17; bars = 200 and 25 μm. The data were derived from four separate samples from pregnant mice. At least, three independent experiments were repeated for this time-point. A total of six samples from pregnant mice were assessed. E, embryo; S, uterine stroma; LE, Luminal epithelium; GE, Glandular epithelium.

For further confirmation the regulation of cyp26a1 to Th17 cells, two experimental mouse models were constructed. Uterine horn–injected anti-cyp26a1 antibody was for the construction of protein function blocking model. Tail vein injection of cyp26a1 lentiviral interference segments was for the construction of RNA interference. Similar to the results of cyp26a1 gene immunization, Th17 cells presented similar trends that increased in peripheral, but reduced in uterus followed by cyp26a1 blocking (Figs 4 and 5). In spite of these blockings on cyp26a1 took on different levels, protein level or gene level, Th17 cells fluctuations are all influenced by the inhibition of cyp26a1.

### At-RA elevated by recombinant plasmid immunization on D5 of pregnancy

**Figure 6:**
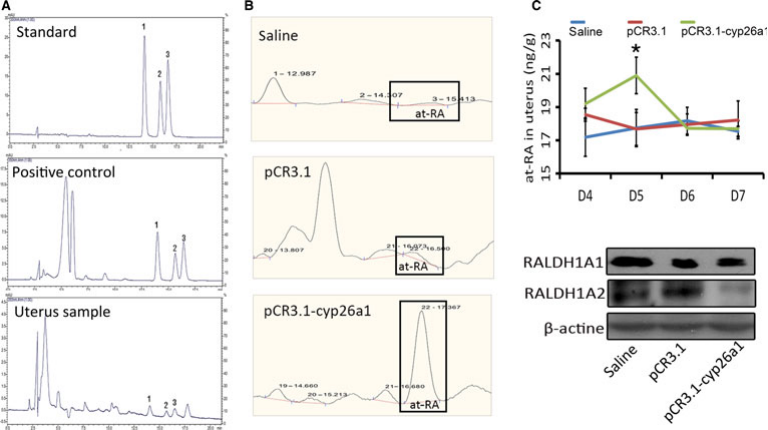
Increase in uterine at-RA level and decrease in its synthase levels during mice peri-implantation following pCR3.1-cyp26a1 immunization, especially on D5 of mice pregnancy. (A) high performance liquid chromatography (HPLC) chromatographs of RA. 1 for 13-cis-RA; 2 for 9-cis-RA; 3 for at-RA. (B) The HPLC chromatograms of D5 uterine at-RA. (C) Uterine at-RA concentration (ng/g) increased in uterus based on uterine at-RA concentration (ng/g) trend graph, under the influence of recombinant plasmid. The uterine at-RA concentration (ng/g) was evaluated by HPLC analysis. Pairwise comparisons were taken between each treatment group, *P < 0.05. At least, five independent experiments were repeated for each time-point. A total of 74 mice were assessed. (D) RALDH1A1 and RALDH1A2 levels were significantly reduced at uterine implantation sites based on Western blotting of pregnancy D5. β-actin was used as a loading control.

Cyp26a1 is major factor in at-RA oxidation [4, 7, 20]. To determine whether at-RA are involved in the regulation of cyp26a1 on Th17 cells in pregnancy, HPLC was employed to measure the RA concentration in the uterus of mice. Using a standard preparation of RA isomers, 13-cis-RA, 9-cis-RA and at-RA were effectively separated at different elution times (Fig. 6A). The elution gradient was 14 min (13-cis-RA)-16.25 min (9-cis-RA)-17 min (at-RA; Fig. 6A). The validity of the RA extraction from tissue was confirmed in Figure 6B. At-RA concentration was stable during peri-implantation in both the saline and pCR3.1 groups (Fig. 6C). However, at D5, a significant increase in the endogenous at-RA concentration was observed in the pCR3.1-cyp26a1–immunized group, resulting in the reduced expression of the two subtypes of retinaldehyde dehydrogenase 1, RALDH1A1 and RALDH1A2 (Fig. 6D). That indicated at-RA would take an important role in cyp26a1 regulation on Th17 cells.

### RARα may be the main at-RA signal transmitter to immune cells in uterus

**Figure 7:**
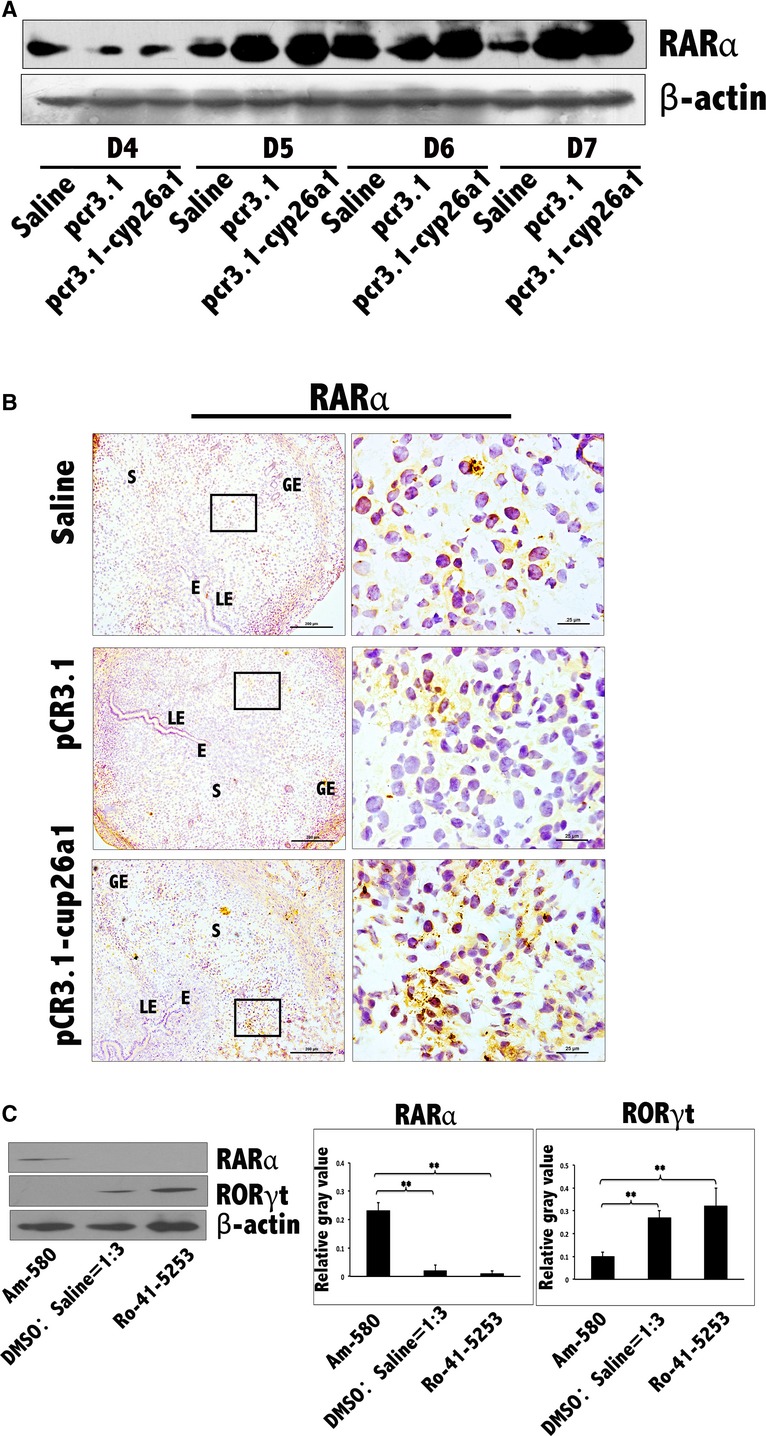
Increase in RARα at uterine implantation sites following pCR3.1-cyp26a1 immunization during mice peri-implantation and further certainty, the influence of RARα on Th17 cells at uterine implantation sites by tail vein injection of RARα specific agonist and antagonist during mice peri-implantation. (A) RARα levels were significantly increased at uterine implantation sites based on Western blotting. β-actin was used as a loading control. (B) RARα levels were obviously increased at implantation sites of D5 pregnancy based on immunohistochemistry following pCR3.1-cyp26a1 immunization; bars = 200 and 25 μm. The data were derived from three separate samples from pregnant mice. At least, three independent experiments were repeated for this time-point. A total of nine samples from pregnant mice were assessed. E, embryo; S, uterine stroma; LE, Luminal epithelium; GE, Glandular epithelium. (C) RARα and RORγt protein expression changes in uterus on D5 of pregnancy in response to administration of agonist (Am-580) and antagonist (Ro-41-5253) of RARα. RARα levels were significantly increased by the activation of Am-580 and significantly reduced by the inhibition of Ro-41-5253 at uterine implantation sites based on western blotting of pregnancy D5. RORγt levels just took the opposite changes on RARα. β-actin was used as a loading control. The administration (125 μg/mouse) of RARα agonist (Am-580) or antagonist (Ro-41-5253) was performed on D3 of pregnancy through tail vein injection; **P < 0.01. Three independent experiments were repeated for this time-point. The data were derived from three separate samples from pregnant mice. A total of nine samples from pregnant mice were assessed.

RA needs its specific receptor to make its biological effects. There are several subtypes of retinoid receptors; significant fluctuations in RARα were observed after immunization of cyp26a1 by the recombinant plasmid (Fig. 7A and B). For further explore the role of RARα as a signal transmitter to immune cells, agonist and antagonist of RARα were administrated. The treatment of RARα agonist Am580 increased the local expression of RARα at the implantation sites of D5 uterus, but with reducing RORγt expression (Fig. 7C). However, the RARα antagonist Ro-41-5253 induced the opposite effects on RARα and RORγt (Fig. 7C). Taken together, these results indicate that RARα might be the delivery of RA signal to downstream immune cells.

## Discussion

In this study, we identified Th17 cells as the important target cell of cyp26a1. They are characterized by specific production of IL-17A, IL-17F and IL-22 as signature cytokines, which are not produced in other Th cells [21, 22]. IL-17A among them, also writing as IL-17, is the main effective cytokine of Th17 cells. Some autoimmune diseases, such as rheumatoid arthritis, multiple sclerosis and chronic inflammatory reactions, are closely associated with overexpression of it. In the light of RORγt directs the differentiation programme of pro-inflammatory IL-17^+^ T-helper cells [23], it is often been taken as the specific marker of Th17 cells. The detection on both of RORγt and IL-17 makes us easy to track Th17 cells in uterus.

Former studies on Th17 cells in mammalian pregnancies suggests that Th17 cells are typically observed in the peripheral blood during the late-phase of normal pregnancy, and increased Th17 cell numbers are detected in abortion samples [24–26]. An increased prevalence of Th17 cells in peripheral blood and the decidua in recurrent spontaneous abortion patients has also been reported [24–25]. The proportion of both Th17 cells and IL-17A concentrations was significantly higher in patients with unexplained recurrent spontaneous abortion (URSA) than in normal early pregnant (NEP) and non-pregnant (NP) patients [27]. The proportion of IL-17A–producing CD4 + T cells among PBLs (peripheral blood lymphocytes) of unexplained RM (recurrent miscarriage) patients is larger than that of healthy cases [28]. These coincide with our observation of Th17 Cell ratio expansion in peripheral blood and spleen followed by cyp26a1 suppression. The frequency of Th17 cells in the uterus is much higher than that in the peripheral blood during pregnancy [24, 26, 29]. Increase in the ratio of uterine Th17 cells needs a process; using chemokine receptor expression profile of CXCR3 as the marker for Th17 cell showed that Th17 cells were nearly absent in first-trimester human decidua [30]. Notably, changes in the numbers of Th17 cells are barely detected in the uteri of missed abortion patients [13]. And the percentage of IL-17A cells in decidua was significantly higher in patients with URSA than in NEP patients [27]. A prominent Th17 response and extensive local inflammation were observed in patients who experience recurrent spontaneous abortions [31]. That is distinguished from the significant reduction in Th17 cells in our study. We believe that it can be result to the different inducing factors and different regulators. Although the data about Th17 role in pregnancy are far from enough, it is reasonable to propose that Th17 cell–prompted inflammation is necessary for successful pregnancy [32–35].

Cyp26a1 is a barrier that blocks RA toxicity and creates an RA gradient for healthy foetal development [36, 37]. Most studies about RA are focused on its function in embryo development. The data about its role in early pregnancy are relatively few. Increase in uterine at-RA on D5 could be obviously reasoned to the cyp26a1 suppression caused by recombinant plasmid immunization. These observations help to explain the Th17 fluctuation observed in both peripheral blood and the uterus and make greater certainty in cyp26a1 regulation on Th17 cells.

Although we have confirmed that Th17 is the downstream target of cyp26a1, the uniqueness of Th17 is still uncertain. In view of the close developmental relevance in Treg (regulatory T cell) and Th17 cell, Treg is also a great potential target. In addition to this, RA synthase RALDH is an important factor of DC (dendritic cell) function [38–39]. As antigen-presenting cells, DCs are so important in the establishment of the immune tolerance microenvironment during healthy pregnancies [41–42]. Considering of this, the relationship between RA and DCs is worth for further study.

RA is a critical signalling molecule that exerts its physiological functions through formation of heterodimers with RARs and RXRs [44]. Multiple isotypes of both the RARs [RARα (NR1B1), RARβ (NR1B2), and RARγ (NR1B3)] and the RXRs [RXRα (NR2B1), RXRβ (NR2B2), and RXRγ (NR2B3)] have been identified. 9-cis-retinoic acid is a bifunctional retinoid activating both RARs and RXRs pathways [45], whereas at-RA activates only the RARs pathway. In this study, RARa induced by at-RA fluctuations was substantially increased in uterus. Research against RARα agonist and antagonist further confirmed the signal significance of RA implantation.

Former studies have shown that the retinoic acid response element (RARE) also exists on cyp26a1, suggesting the regulation of RA signalling [46]. Thus, a feedback loop would exist between RA and cyp26a1, which presented a new clue of research.

In summary, observations in our research suggested that RA is an essential mediator in cyp26a1 regulation and Th17 cells are an important Th cell subtype of cyp26a1 targeting in mice peri-implantation.

These results might be beneficial for further understanding the signal pathway underlying Th cell differentiation and maintenance and might also have implications in the immunotherapy. Furthermore, this research could provide insights into the involvement of Th cells in clinical diseases, such as tumours and several gynaecological conditions, including preeclampsia, ectopic pregnancy, endometrial cancers and recurrent miscarriages. We hope that this basic laboratory work will lead to more alarm regarding the utilization of RA as a therapeutic in the clinic. And, we also wish the effectiveness of cyp26a1 gene vaccine could inspire relevant researches for its potential medicinal value.
